# Short-term effects of the “I Spy Feelings” program on emotion regulation in 5- to 6-year-old children

**DOI:** 10.3389/fpsyg.2023.1016521

**Published:** 2023-08-03

**Authors:** Annaliese Arthur, Maryanne McDevitt, Rosanna M. Rooney, Amber MacLeod, Robert T. Kane, Kate Tonta, Kaitlin McMillan, Jacob Peckover, Natalie Baughman

**Affiliations:** ^1^Discipline of Psychology, School of Population Health, Curtin University, Bentley, WA, Australia; ^2^Curtin enAble Institute, Curtin University, Bentley, WA, Australia

**Keywords:** emotion regulation, early intervention, early childhood, prevention program, emotional coping, internalizing disorders, externalizing disorders

## Abstract

**Introduction:**

Mental health difficulties in early childhood can have a debilitating and ongoing impact throughout an individual’s life; emotion regulation can serve as a protective factor. Therefore, evidence-based prevention programs that teach children effective skills and strategies for emotion regulation are needed.

**Methods:**

As part of the Aussie Optimism pilot study evaluating the “I Spy Feelings” program, this study aims to assess the short-term effects of the program on emotion regulation in pre-primary aged children after 2 months via a longitudinal cluster randomized controlled trial. Participants included parents (*N* = 73) of 5- to 6-year-old children attending four different Catholic primary schools. Children from two of the schools were allocated to the intervention group where they participated in the program (*N* = 33), while children from the other two schools were allocated to the control group where they did not (*N* = 40). At each time point, all parents completed abridged Children’s Emotional Management Scales measuring how well parents believe their child is able to cope with anger, sadness and worry.

**Results:**

A significant intervention effect 2 months after intervention was found for the outcome of anger coping such that parents whose children were in the intervention group reported significantly greater improvement in their children’s ability to cope with anger compared to parents whose children were in the control group. No significant effect was found for the outcome of sadness, and results for the worry subscale were inconclusive due to unacceptable internal consistency.

**Discussion:**

The present study provides insight into the benefit of programs designed to enhance the emotion regulation skills of very young children. Further follow-up is needed to assess whether the “I Spy Feelings” program has lasting effects.

## Introduction

Research has found that children as young as 4 years old can experience mental disorders, which if not addressed may persist throughout the lifespan (Lawrence et al., 2015). A review conducted by Vasileva et al. (2021) of the international prevalence of mental disorders in children aged 1–7 found prevalence rates of 8.5% and 1.1% for anxiety and depressive disorders, respectively. Furthermore, comorbid presentations of anxiety and depression are common in children and adolescents (Melton et al., 2016). Poor mental health can adversely affect children’s development at critical stages in life, and have lasting detrimental effects (Norona and Baker, 2017). Mental health difficulties can negatively impact children’s relationships with peers and family as well as academic performance and success (Scott et al., 2016; Norona and Baker, 2017). Children with anxiety and/or depression often experience low mood, feelings of hopelessness, and worry, which can subsequently develop into suicide ideation (Huberty, 2012). However, there currently exist a number of evidence-based skills and strategies that children can learn to improve their current and future mental wellbeing (García-Carrión et al., 2019). One such skill is the ability to regulate emotions.

Emotion regulation is a flexible and individualized skill that involves correctly identifying, evaluating and modifying emotions to facilitate rather than hinder functioning in particular situations (Doyle and Sullivan, 2015; Maggio et al., 2016; Hipson et al., 2017; Malhi et al., 2017). There are a number of strategies that can be used to regulate one’s emotions, though not all of these strategies are equally effective (Naragon-Gainey et al., 2018). Some strategies, such as avoidance, rumination, and suppression, may be maladaptive. Avoidance refers to the tendency to keep away from situations or stimuli which trigger negative emotions (Hofmann and Hay, 2018). Rumination describes the process of focusing one’s attention on all possible consequences of a feared or distressing situation, as well as arising emotions and thoughts (Aker et al., 2014). Suppression refers to the conscious process of pushing unwanted negative emotions or negative emotion-provoking thoughts out of awareness (Ciuluvica et al., 2019). While avoidance, rumination, and suppression strategies may be effective for regulating and reducing stress in the short-term, they have been found to increase involuntary arousal and cognitive load in the long-term, and thus can lead to emotion dysregulation (Berking and Whitley, 2014; Inwood and Ferrari, 2018). Dysregulation of emotions such as sadness, worry and anger can subsequently contribute to internalizing disorders such as anxiety and depression (Folk et al., 2014; Abdi and Pak, 2019).

Other strategies for emotion regulation such as attentional redeployment, problem solving, and cognitive reappraisal can be referred to more specifically as “emotional coping strategies,” as they involve conscious attempts to reduce negative emotional responses associated with stress (Skinner and Zimmer-Gembeck, 2007). Attentional deployment involves refocusing attention to different (perhaps more positive) aspects of a situation (Torrence and Connelly, 2019). Problem solving is a mental process that involves discovering, analyzing, and solving problems which are contributing to negative emotions (Aldao and Nolen-Hoeksema, 2010). Cognitive reappraisal strategies aim to modify the emotional meaning and impact of situations which elicit emotions (Gross and John, 2003). There is a small amount of neuropsychological evidence to suggest children aged 4–7 can use cognitive reappraisal but only with specific external guidance and support (Hua et al., 2015). Findings suggest that emotional coping strategies may be more adaptive for emotional regulation and more effective at protecting an individual’s mental health and wellbeing than strategies such as rumination and suppression (Aldao and Nolen-Hoeksema, 2010; Naragon-Gainey et al., 2018). According to the literature, regulating emotions using emotional coping strategies leads to individuals becoming more resilient, flexible, patient, and optimistic when faced with an emotional difficulty (Ogelman and Onder, 2019).

### Influences on emotion regulation strategies

Emotion regulation difficulties can begin in childhood and lead to psychological instability, impaired mental health and a lower level of life satisfaction and happiness (Saxena et al., 2011; Perry et al., 2016). In order to prevent children from experiencing emotional dysregulation and/or developing mental disorders such as anxiety and depression, it is important to understand the factors which determine whether children learn to regulate their emotions using adaptive emotional coping strategies or using maladaptive strategies such as avoidance, rumination, and suppression.

All past experiences and contexts shape an individual’s ability to regulate their emotions, as well as the processes that they use to do so (Aldao et al., 2015). Research has found parenting styles to have a significant influence on children’s emotion regulation strategies (Norona and Baker, 2017). Emotion regulation in very young children involves seeking assistance and guidance for emotional support and knowledge to identify and understand their emotions (Norona and Baker, 2017). If a child’s caregiver is seemingly emotionally unavailable to the child, the child’s capacity to develop adaptive emotional regulation strategies may be hindered (Norona and Baker, 2017). Alternatively, if a child’s caregiver is emotionally responsive, supports the child in regulating their emotions, and can model adaptive emotional coping strategies, the child is more likely to learn and use these adaptive strategies themselves (Caiozzo et al., 2018). The experience of trauma early in life may also impact children’s emotional regulation strategies *via* the formation of unhelpful self-beliefs (Caiozzo et al., 2018). These beliefs can influence information processing and subsequent emotional reactions, increasing the child’s likelihood of using maladaptive emotion regulation strategies in response to stressful events (Dozois et al., 2008; Dadomo et al., 2016). Indeed, research has found a significant negative relationship between the ability to regulate emotions and negative past experiences (Aldao et al., 2015; Hipson et al., 2017).

One theory which focuses on the development of emotional coping throughout childhood is the “families of coping” theory (Skinner and Zimmer-Gembeck, 2007). This theory involves the postulation that while similar emotion regulation strategies are used at each stage of a child’s development, the presentation of these strategies changes with the child’s growing knowledge on the strategy and how to use it (Skinner and Zimmer-Gembeck, 2007). For example, an infant practicing attentional deployment may do so by physically turning their head toward preferred stimuli, while an older child may be more likely to use cognitive strategies to achieve this same strategy for emotional regulation (Skinner and Zimmer-Gembeck, 2007). In early childhood, the capacity for independent regulation increases dramatically, as does the repertoire of strategies available to the child (Compas et al., 2017). According to “families of coping” theorists, the development of adaptive emotional coping strategies in early childhood is crucial for ensuring that healthy habits are practiced and maintained throughout the lifespan (Wakschlag et al., 2019).

### Emotion regulation programs

Early childhood is the most malleable neurodevelopmental period, meaning it will be easier for children to learn adaptive emotional coping strategies during this period rather than later in childhood (Wakschlag et al., 2019). There is limited literature on evaluations of emotion regulation programs for very young children. However, the literature that is available includes significant intervention effects for group programs that teach age-appropriate skills and strategies to improve social and emotional competence (Izard et al., 2004; Domitrovich et al., 2007; Bradley et al., 2012). For example, ‘I Can Problem Solve’ is a universal, school-based prevention program which teaches problem solving skills to 5–6-year-old children using puppets as an engaging and age-appropriate medium (Anliak and Sahin, 2010). This program has been found to significantly reduce inhibited and aggressive behaviors in children who took part in the intervention (Anliak and Sahin, 2010). Programs like ‘I Can Problem Solve’ improve the emotional coping capabilities of young children, which may in turn reduce the prevalence of internalizing disorders such as anxiety and depression (Ogelman and Onder, 2019).

Some emotion regulation programs, such as the “Early HeartSmarts Program,” are targeted to children from ethnic minority or lower socioeconomic backgrounds (Izard et al., 2004; Bradley et al., 2012). Programs which are targeted to certain demographic characteristics may be useful for these specific subgroups, but have reduced external generalizability and do not provide insight into the effectiveness of mental health programs for the whole population (Zulman et al., 2008). Universal programs are beneficial as they ensure that high-risk children are not overlooked, and also avoid any impacts of stigma surrounding mental illness (Ahlen et al., 2015). Additionally, most emotion regulation programs are implemented within the school context, and some evaluations do not take into account the clustering limitations of the data (Domitrovich et al., 2007). Therefore, it is important that emotion regulation programs are assessed using analyses, such as general linear mixed model (GLMM) analysis, which adhere to the intra-school dependencies (i.e., the non-independence of the data as a result of students nested in classrooms). Studies which assess mental health programs developed by Aussie Optimism often use GLMM for evaluation (Oorloff et al., 2021).

### Aussie Optimism

Aussie Optimism programs (AOPs) are universal programs implemented in a classroom setting which aim to improve mental wellbeing in children (Tennant et al., 2017). Generally, AOPs are designed to be delivered in 1 hour timeslots across 10 sessions spanning one school term (Tennant et al., 2017). The AOPs teach self-regulation, facing fears, and relaxation as strategies to improve emotional competence (Pophillat et al., 2016). The AOPs have established significant intervention effects regarding the reduction of anxiety, depression and suicidality, as well as increases in psychosocial protective factors (Rooney et al., 2013; Roberts et al., 2018). The outcomes of AOPs focus on social and emotional competence which constitute protective factors against poor mental health (Domitrovich et al., 2017). Social competence involves the building and maintaining of healthy relationships with peers (Ryan and Shim, 2016), while emotional competence involves adaptive emotion awareness, expression, and regulation (Buckley et al., 2003). While AOPs teach skills and strategies which should improve children’s capacity for emotional coping, outcomes regarding emotion regulation have not yet been specifically assessed. Furthermore, although AOPs were previously tailored only to children aged 6 years or older, recent studies have found that earlier intervention is beneficial, as the skills and strategies learnt can be maintained and improved upon as children move into adolescence and adulthood (Wakschlag et al., 2019). These findings supported the development of an AOP for pre-schoolers.

The “I Spy Feelings” program developed by Aussie Optimism aims to improve social and emotional competence in pre-primary aged children (4-to 6-years old), with emotion regulation strategies as a key component (Oorloff et al., 2021). The program teaches children how to identify, understand, and regulate their emotions through strategies such as deep breathing and muscle relaxation techniques (Oorloff et al., 2021). A study assessing the immediate effects of this program on emotion regulation found a significant intervention effect such that children who participated in the program had an enhanced capacity for regulating the emotion of anger (Oorloff et al., 2021). This study therefore demonstrated that emotion regulation prevention programs are effective for very young children. However, Oorloff et al. (2021) found no intervention effect regarding children’s ability to regulate emotions of sadness or worry immediately after the intervention was delivered. The short-term effects of the “I Spy Feelings” program have not yet been evaluated. In the current study, short-term effects refer to those present 2 months after completing the program. It is important to investigate the short-term effects to ensure the effectiveness of the program in enhancing children’s use of strategies for regulating their anger is maintained, and also explore whether the skills that children learn in the program develop over time with practice.

### Rationale for the current study

A synthesis of the existing literature suggests that adaptive strategies for emotion regulation act as a protective factor against internalizing disorders such as anxiety and depression (Aldao and Nolen-Hoeksema, 2010; Naragon-Gainey et al., 2018). It is important to implement emotion regulation programs in school starting in early childhood as this is the most malleable neurodevelopmental period and not all children have a positive emotional climate at home where emotion regulation is adequately taught (Norona and Baker, 2017). Universal prevention programs are beneficial as they reduce the issue of stigma surrounding mental illness, and also eliminate the possibility of over-looking children who are at high risk of developing mental disorders (Ahlen et al., 2015). There currently exists a gap in the literature regarding the short-term follow-up effects of universal prevention programs implemented in early childhood on children’s use of adaptive emotional regulation strategies.

The aim of the present study was to assess the short-term effects of the “I Spy Feelings” program on emotion regulation in children aged 5- to 6-years. Emotional coping was assessed through parent reports of their child’s capacity to regulate feelings of anger, sadness, and worry. It is hypothesized that children who complete the “I Spy Feelings” program will exhibit a significantly greater improvement at 2 months post-intervention in their ability to cope with anger (H1a), sadness (H1b), and worry (H1c) compared to children who do not participate in the program.

## Method

### Research design

The current research constitutes part of a larger Aussie Optimism study testing the efficacy of the “I Spy Feelings” program. While the larger study involves the collection of data from children, parents and teachers, the present study focused on measures of emotional regulation as reported by parents. It utilized a longitudinal (pre-test, post-test, 2 month follow-up) cluster randomized control trial. Initial data was collected during term 3 (July–September) of 2019. Four Perth Catholic primary schools were randomly allocated to either the “I Spy Feelings” intervention condition or a waitlist control condition, such that there were two schools in each condition. Schools in each condition were matched on the basis of socioeconomic status and size. The socioeconomic status of the schools was determined using the Department of Education H-Index (Department of Education, 2003). The H-Index provides a value of 1–10, with 1 being the least socioeconomically disadvantaged, and 10 being the most disadvantaged.

The independent variable was delivery of the “I Spy Feelings” intervention, with school allocation to the intervention or control group determining children’s exposure. The dependent variables were children’s emotional coping scores regarding the emotions of anger, sadness, and worry. The primary outcome of this study was the emotional coping of children as reported by their parents.

### Participants

**Figure 1:**
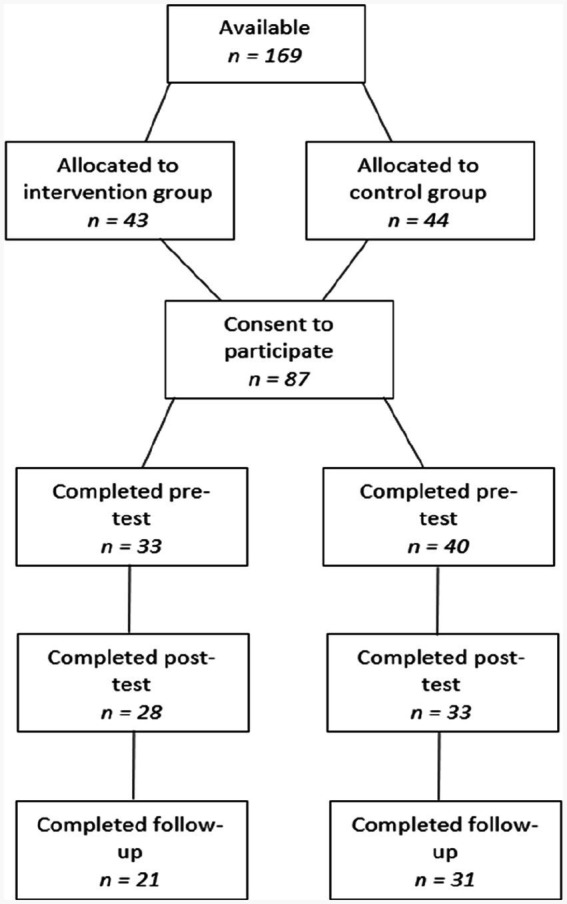
CONSORT participant diagram.

Participants (*N* = 73) included parents of children (one parent per child) from 4 Catholic primary schools around the Perth metropolitan area. In order take part in the “I Spy Feelings” program, children had to be fluent in English and aged between 4 to 6 years. The mean age of the children included in the program was 5 years, and 65.8% (48) were female. Further demographic information including children’s ethnic origin, previous mental health difficulties, and family financial situation has been published elsewhere (Oorloff et al., 2021). Participant attrition throughout the study is represented within a CONSORT (Consolidated Standards of Reporting Trials) diagram in Figure 1. Within the intervention group, there was a 16% attrition rate between pre-test and post-test, and a 25% attrition rate between post-test and 2 month follow-up. Within the control group, there was a 17% attrition rate between pre-test and post-test, and a 6% attrition rate between post-test and follow-up. Reasons for attrition may include lack of interest from parents or forgetting to return the questionnaires.

The present study used general linear mixed model (GLMM) analyses to determine if there were significant differences in the extent to which children in the intervention condition compared to the control condition displayed change in their emotional coping. There were two major reasons for using GLMM analyses in the present study. First, GLMM analyses are “generalised” as they can accommodate variables which do not have normal distributions (which was the case in the present study where most outcomes had a positive skew). Second, participant attrition was an issue in the present study due to its longitudinal nature. GLMM analyses are less sensitive to attrition as they do not require data from participants at each time point. GLMM analyses use a maximum likelihood procedure, which uses all data present at each time point, therefore participants do not have to provide data for every time point to be included in the analysis. Consequently, there is no need to replace whole case missing data. The maximum likelihood procedure also eliminates the need to compare completers with non-completers as well as concerns around sampling bias.

Within the present study, three GLMM analyses were conducted to see if there was a change in either sadness, anger, or worry scores, respectively. Each of the three analyses included two nominal random effects (student and class), one nominal fixed effect (group: intervention or control), one ordinal fixed effect (time: pre-test and follow-up), and the 2-way group × time interaction. The three GLMM analyses were implemented through SPSS’s (Version 25) GENLINMIXED procedure.

Anger, sadness, and worry are conceptualized as separate, yet related constructs and each analysis was hypothesis driven. This allowed the GLMM analyses to test the effect of the intervention on each construct separately and as such removed the need to adjust the alpha value for multiple comparisons, similar to what has been done in previous research (Cheng et al., 2018).

G*Power (Version 3.1.9.2) estimated that a sample size of 69 was required for 80% probability of capturing a “large” between-group difference on the outcome at follow-up. This was after controlling for any between-group differences at pre-test. However, the design effect calculation needed to account for the reduction in effective sample size due to intra-school clustering in the data.


Design effect=1+(m−1)×ICC

$$2.28=1+\left(17–1\right)\times 0.08$$

where *m* (=17) represents the average number in each of the 4 schools (69/4 = 17). The ICC (=0.08) is the predicted intra-class correlation for the CEM subscales across the four schools. The design effect number of 2.28 needs to be multiplied to get intended sample size, which equates to be 157 (39 students in each school). The data for this study only included 73 participants beginning at pre-test. According to the *a priori* power analysis the sample size is considered weak. However, the initial study conducted at post-test obtained significant intervention effects with this sample size (Oorloff et al., 2021).

### Measures

#### The Children’s Emotional Management Scales (CEMS; Zeman et al., 2001)

The Children’s Emotional Management Scales assess children’s emotional regulation strategies in response to three key emotions: anger, sadness, and worry. Each scale comprises three subscales: inhibition strategies, dysregulation strategies, and coping strategies. Thus, in order to assess children’s capacity for emotional coping at each time point, the present study utilized only the items from the coping subscales of the CEMS. This included 4 items assessing strategies for coping with anger (e.g., “when my child is feeling mad, he/she can control his/her temper”), 5 items assessing children’s strategies for coping with sadness (e.g., “my child can stop him/herself from losing control of his/her sad feelings”), and 3 items assessing strategies for coping with worry (e.g., “my child talks to someone until he/she feels better when he/she is worried”). The score range for the anger coping scale was 4–12, for the sadness scale the score range was 5–15, and finally the score range for the worry coping scale was 3–9. Items were rephrased to allow parents to report their perceptions of their child’s use of emotion regulation strategies. For each item, parents reported the frequency with which their child uses the described strategy on a three-point Likert scale comprising response options of 1 (*hardly ever*), 2 (*sometimes*), and 3 (*often*). Thus, higher scores on the abridged CEMS indicate higher capacity for emotional coping regarding anger, sadness, and worry.

The CEMS has consistently displayed acceptable internal consistency and test re-test reliability (Sanders et al., 2015). Research has also established discriminant validity for this scale (Zeman et al., 2010). The abridged CEMS used in the present study demonstrated internal consistency values of 0.81, 0.64, and 0.38 for the anger, sadness, and worry coping scales, respectively. Internal consistency of 0.7 or higher is generally considered to be valid for research purposes (Cronbach, 1951). As there are only 4 or 5 items in the anger and sadness coping scales, the internal consistency of these scales can be considered sound (Loewenthal, 2001). However, the internal consistency for the worry coping scale presents a limitation of the present research. Oorloff et al. (2021) found no such issue with the internal consistency of the worry coping scale.

#### Demographic questionnaire

Demographic information regarding age, gender, socioeconomic status, and any mental health issues experienced within the past 12 months was collected from parents using a demographic questionnaire administered at pre-test.

### Intervention

The “I Spy Feelings” program consists of 10 sessions which are designed to run for 40 min each. The topics covered by each session are outlined in Table 1. In the current study, teachers implemented the program with their students in their usual classroom, covering 2 sessions weekly for 5 weeks. It was initially intended for the program to run over 10 weeks, however delays in ethical clearance resulted in a condensed program. The program emphasizes language around emotion, problem-solving, interpersonal skills, and self-management skills. The play-based activities in the program are age-appropriate, including stories and songs intended to develop children’s social, emotional, and cognitive abilities. The program is intended to be delivered universally within the classroom; however, some students may be identified as exhibiting additional difficulties that extend beyond the scope of the program. These students can be referred on through pastoral care or school psychology pathways that exist within the school.

**Table 1:** Modules in the “I Spy Feelings” program.

Module	Name	Description
1	Feelings	Learning of group rules, listening to others and identifying emotions
2	Feeling happy	Identifying body cues for feeling happy
3	Language for problem solving 1	Develop emotion vocabulary, cooperative play and following rules
4	Language for problem solving 2	Develop language to sequence and generate alternatives
5	Feeling sad	Develop language and body cues for feeling sad
6	Coping with sadness	Develop coping strategies for feeling sad
7	Feeling scared or worried	Develop language and body cues for feeling scared and worried
8	Coping with fear and worry	Develop coping strategies for feeling scared and worried
9	Feeling angry	Develop language and body cues for feeling angry
10	Coping with anger	Develop coping strategies for feeling angry

### Procedure

Before research began, ethics approval was obtained from Curtin University’s Human Research Ethics Committee (HREC), and approval was obtained from the Catholic Education office of Western Australia (CEWA). All CEWA schools with pre-primary classes within the Perth Metropolitan area were approached (*N* = 141) and any schools who expressed interest were matched into pairs on the basis of socioeconomic status and size. Four schools (one pair with a high socioeconomic status and one pair with a low socioeconomic status) were then randomly selected to take part in the study. Following this, one school from each pair was then randomly allocated to the intervention condition and the other to the control condition, so that two schools were allocated to each condition. Once the school principals gave their consent for their schools to take part in the project, participants were recruited. Information letters and consent forms were distributed to the parents and children in both the control and intervention groups and later collected. After consent and ethics approval were obtained, teachers were trained in the program and its implementation in the classroom by Aussie Optimism staff *via* a 4 h training workshop.

Prior to delivery of the intervention, parents completed the CEMS (Zeman et al., 2001) to assess their child’s use of emotional coping strategies during week 1 of term 3. This pre-test data collection continued for 3 weeks due to absentees and participants who consented later in the term. After all pre-test questionnaires were collected, the “I Spy Feelings” program commenced within the classrooms allocated to the intervention condition. Two modules were implemented per week, resulting in the completion of the program by week 8 of term 3. Children allocated to the control condition completed lessons as per the usual curriculum during this time. After the program was completed, parents were asked to complete the CEMS (Zeman et al., 2001) again to assess their child’s use of emotional coping strategies. This post-test data was mostly collected during week 9 of term 3. However, some participants took up to 5 weeks to return their post-test questionnaire. Two months after the conclusion of the program, the CEMS (Zeman et al., 2001) was re-sent to the parents to assess the short-term effects of the “I Spy Feelings” program on the children’s use of emotional coping strategies. Children who were initially allocated to the control condition were then given the opportunity to take part in the program.

The data from the CEMS questionnaires collected at pre-test, post-test, and 2 month follow-up was entered into Qualtrics by five people in the Aussie Optimism research team. The Aussie Optimism team consisted of trained volunteers as well as Honors and Masters students. Each questionnaire was cross-scored by two people in the team to reduce error.

## Results

Table 2 contains the descriptive statistics for each of the emotional coping scales over time. Table 3 displays the results of the three GLMM analyses conducted.

**Table 2:** Mean emotional coping scores for each group over time.

Emotional coping scale	Time	Group	Mean score	Std. error	95%	CI
					LL	UL
Anger coping scale	Pre-test	Control	7.67	0.33	7.02	8.32
		Intervention	7.63	0.36	6.93	8.33
	Follow-up	Control	7.44	0.36	6.74	8.15
		Intervention	8.59	0.40	7.80	9.39
Sadness coping scale	Pre-test	Control	9.51	0.52	8.48	10.54
		Intervention	9.37	0.53	8.33	10.42
	Follow-up	Control	9.47	0.71	8.07	10.87
		Intervention	9.74	0.67	8.43	11.05
Worry coping scale	Pre-test	Control	7.04	0.11	6.82	7.26
		Intervention	6.84	0.13	6.59	7.09
	Follow-up	Control	6.89	0.21	6.48	7.31
		Intervention	7.35	0.26	6.83	7.86

CI, confidence interval; LL, lower limit; UL, upper limit.

**Table 3:** Results of GLMM analyses for anger, sadness, and worry coping scales.

	Anger		Sadness		Worry	
Source	*F*(2, 186)	Sig.	*F*(2, 185)	Sig.	*F*(2, 185)	Sig.
Corrected model	2.248	0.051	0.145	0.981	1.224	0.296
Time	2.374	0.096	0.083	0.921	130.820	0.000
Group	1.308	0.254	0.032	0.858	0.226	0.635
Group × time	3.535	0.031	0.233	0.729	83.252	0.000

**Figure 2:**
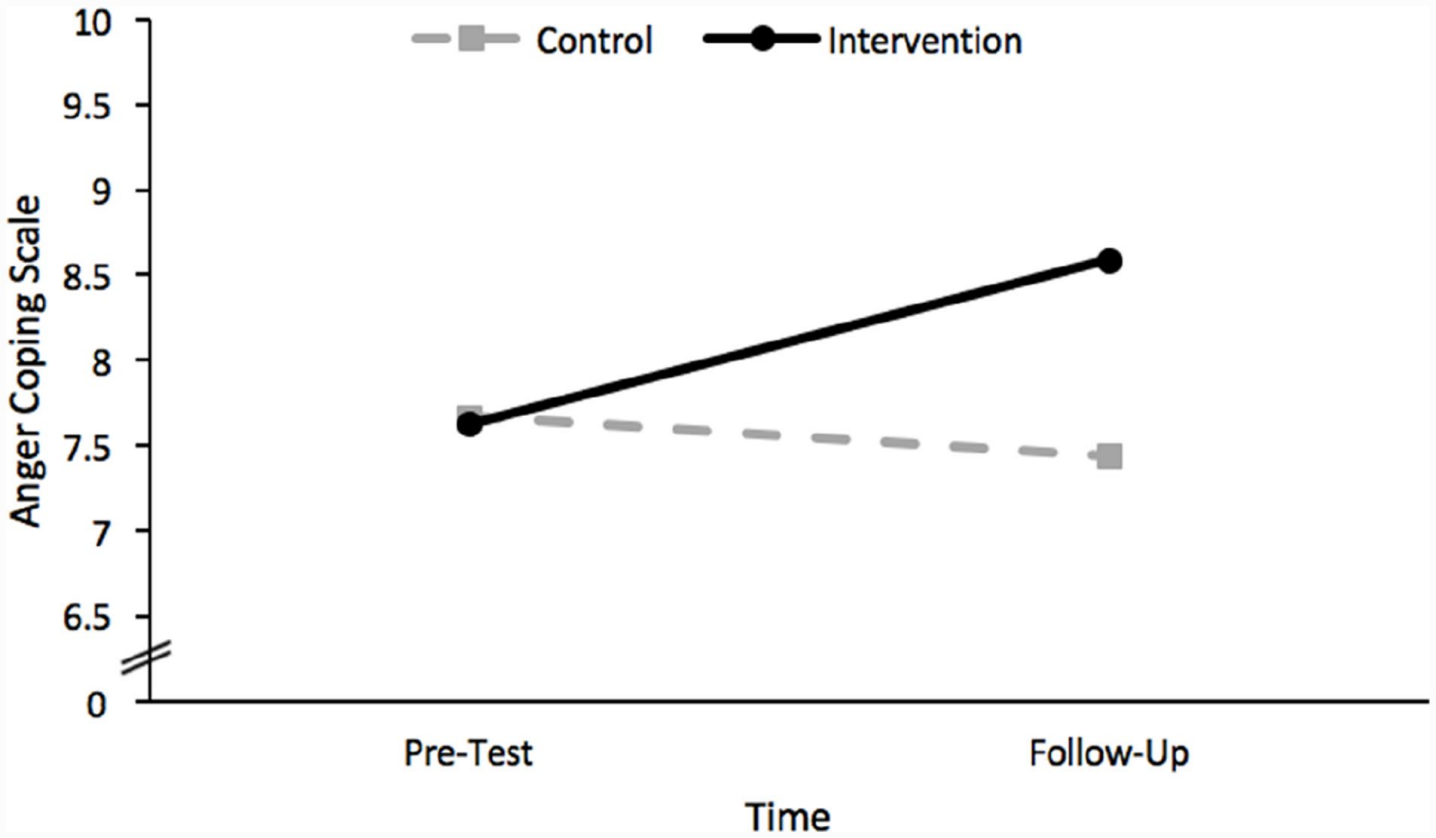
A plot of the significant interaction effect between group and time revealed by the GLMM conducted using anger coping scores as the dependent variable.

Considering first the results of the analysis which included scores on the anger coping scale as the dependent variable, neither the main effect of time, *F*(2, 186) = 2.37, *p* = 0.096, nor the main effect of group, *F*(2, 186) = 1.31, *p* = 0.254, were significant. This analysis did reveal a statistically significant interaction effect between group and time, *F*(2, 186) = 3.54, *p* = 0.031. Therefore, this interaction indicates that the effect of time differed for each group, as depicted in Figure 2. The nature of this interaction was revealed using simple main effect analyses. Specifically, there was a significant simple main effect of time for the intervention group such that the anger coping scores of children in this group significantly increased between pre-test and follow-up, *t*(186) = 2.80, *p* = 0.006. There was no significant simple main effect of time for the control group, *t*(186) = 0.30, *p* = 0.763.

**Figure 3:**
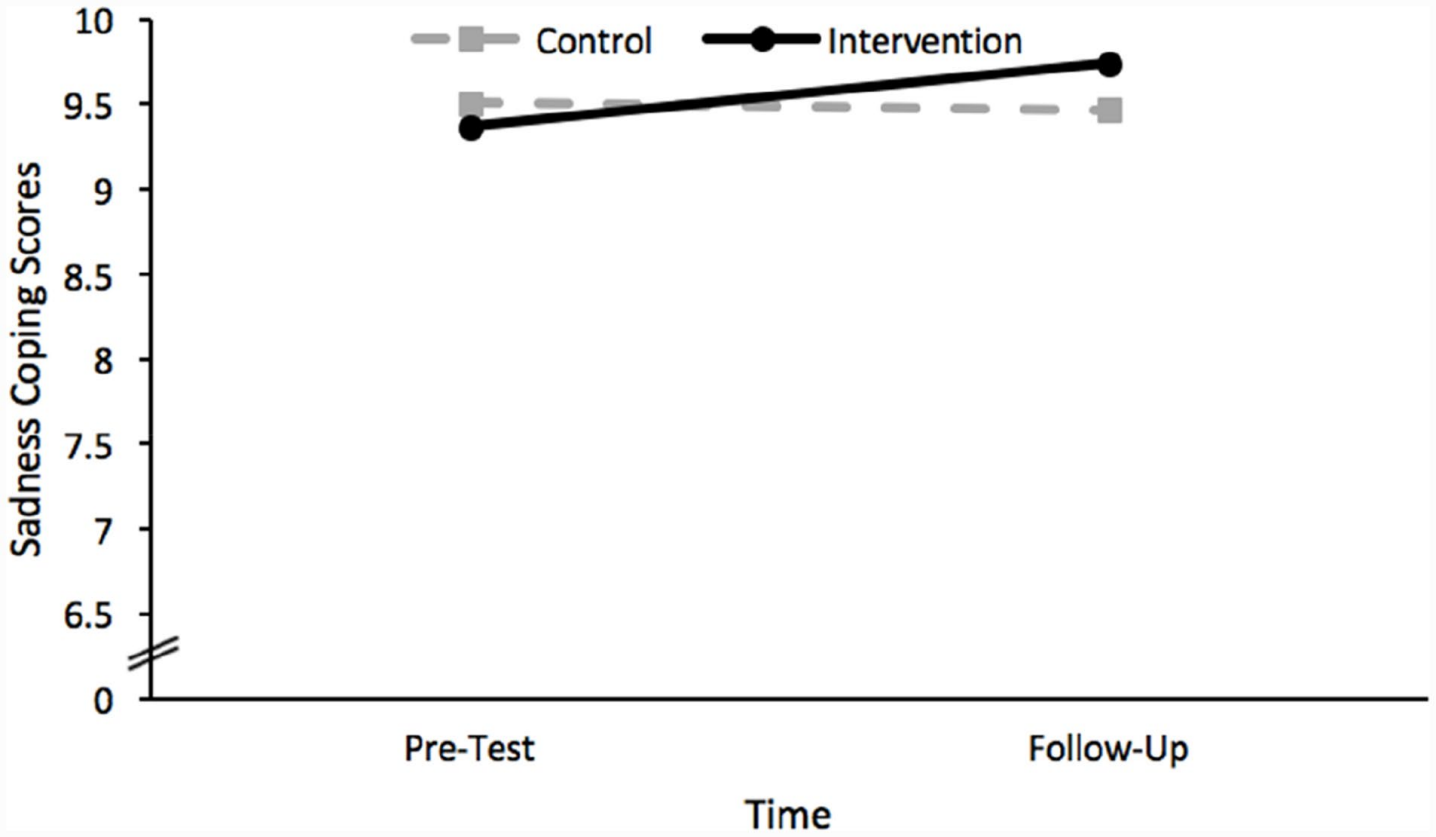
A plot of the non-significant interaction effect between group and time revealed by the GLMM conducted using sadness coping scores as the dependent variable.

Considering next the results of the analysis which included scores on the sadness coping scale as the dependent variable, the analysis revealed no significant effects. Specifically, neither the main effect of time, *F*(2, 185) = 0.08, *p* = 0.921, the main effect of group, *F*(2, 185) = 0.03, *p* = 0.858, nor the group × time interaction effect, *F*(2, 185) = 0.02, *p* = 0.792 achieved significance. The lack of a statistically significant interaction effect indicates that the effect of time did not significantly differ for each group, as depicted in Figure 3.

**Figure 4:**
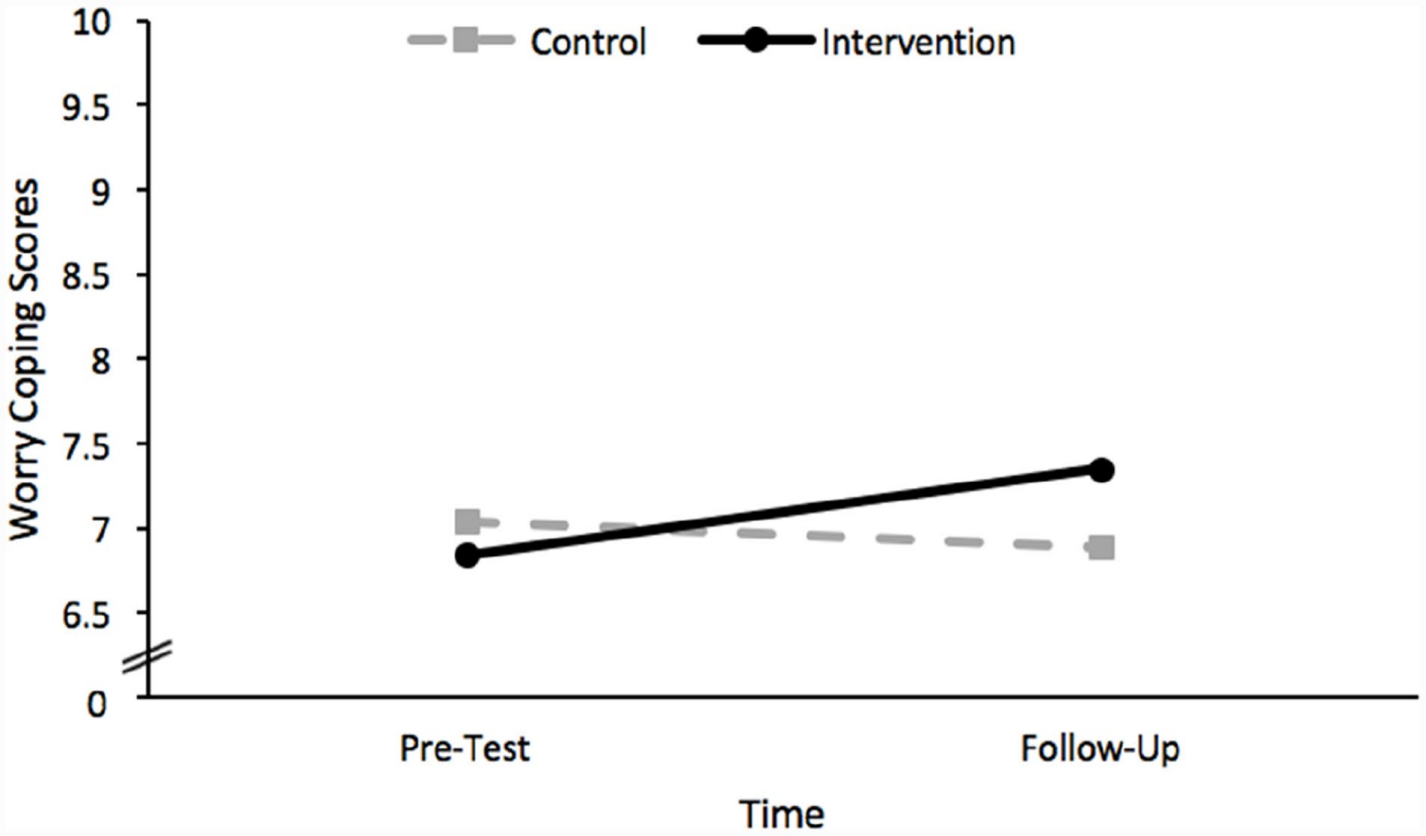
A plot of the significant interaction effect between group and time revealed by the GLMM conducted using worry coping scores as the dependent variable.

Finally, considering the results of the analysis which included scores on the worry coping scale as the dependent variable, the main effect of group was not significant, *F*(2, 185) = 0.23, *p* = 0.635. The main effect of time was significant, *F*(2, 185) = 130.82, *p* < 0.001, but was subsumed within the statistically significant interaction effect between group and time, *F*(2, 185) = 83.25, *p* < 0.001. This interaction indicates that the effect of time differed for each group, as depicted in Figure 4. The nature of this interaction was revealed using simple main effect analyses. Specifically, there was a significant simple main effect of time for the intervention group such that the worry coping scores of children in this group significantly increased between pre-test and follow up, *t*(185) = −3.82, *p* < 0.001. There was no significant simple main effect of time for the control group, *t*(185) = 1.45, *p* = 0.143.

## Discussion

The purpose of the present study was to assess the short-term effects of the “I Spy Feelings” program on emotion regulation in children aged 5- to 6-years. Results partially support the hypothesis that children who complete the “I Spy Feelings” program will exhibit a significantly greater improvement in their ability to cope with various emotions compared to children who do not participate in the program. Specifically, the present study found evidence to suggest that 2 months after completing the “I Spy Feelings” program, children in the intervention group showed significantly greater improvements in their ability to cope with anger (H1a) than children in the control group, but not sadness (H1b). The results also suggest that children who received the intervention demonstrated greater improvements in their ability to cope with worry (H1c) than those who did not receive the intervention. However, this finding should be considered with caution, given the low internal consistency of this subscale in the present data. The implications of each of these findings will be discussed, before limitations of the present study are identified and suggestions offered concerning how future studies might build upon and extend the present research.

First, consider the present finding regarding children in the intervention group’s enhanced capacity for coping with anger compared to those in the control group. These results provide empirical support to previous research which has investigated the capacity for prevention programs to teach adaptive emotional regulation strategies to children in preschool (Izard et al., 2004; Domitrovich et al., 2007; Anliak and Sahin, 2010; Bradley et al., 2012). Importantly, this finding mirrors those obtained by Oorloff et al. (2021) when they assessed the immediate effects of the “I Spy Feelings” program on emotion regulation. Thus, the present research demonstrates that the significant positive effects of this program on children’s ability to cope with the emotion of anger are maintained 2 months after delivery of the intervention. According to previous research, this finding may have flow-on implications regarding the impact of the “I Spy Feelings” program on children’s broader mental health and wellbeing (Ogelman and Onder, 2019). Specifically, it may be that early development of these adaptive emotional coping strategies within the program serves as a protective factor against the onset of mental disorders later in life (Aldao and Nolen-Hoeksema, 2010; Naragon-Gainey et al., 2018). Further research is necessary to confirm whether the “I Spy Feelings” program is associated with subsequent lower rates of mental disorders.

Next, consider the present finding regarding children in the intervention group’s enhanced capacity for coping with worry compared to those in the control group. This finding contrasts the results of Oorloff et al.’s (2021) initial study, which reported no significant differences in the extent to which worry coping was improved for children in the intervention group compared to those in the control group. Therefore, the present study’s significant finding may reflect the impact of skills practice that children engaged in during the 2 months between the conclusion of the program and the collection of follow-up assessment data. As with all skills, the strategies for emotion regulation taught within Aussie Optimism programs (AOPs) require ongoing practice in order for children to become proficient (Roberts et al., 2018). It may be that the strategies that children were taught to use to cope with worry simply require more practice in order to be effective than those that they were taught to use to cope with anger. Thus, while improvements in worry coping were not evident immediately after the program, they eventually became apparent following 2 months of practice. Thus, the present finding of significantly greater improvements in worry coping for children in the intervention group compared to those in the control group highlights the need for follow-up studies when investigating the efficacy of prevention programs. As stated previously, the present findings regarding worry coping must be interpreted with caution due to the worry coping scale’s low internal consistency. This constitutes a significant limitation of the present study, and will be further discussed below.

Finally, consider the present outcome of no significant difference between children in the intervention group and the control group regarding change in their capacity for coping with sadness. There exist a number of potential explanations for this outcome. As previously discussed, the emotional coping strategies taught within the “I Spy Feelings” program must be practiced in order to be effective (Roberts et al., 2018). Therefore, one possibility is that the skills that children were taught to use in order to cope with sadness require more practice to be effective than is generally possible within a timeframe of 2 months. It may be that with a longer follow-up period, the children would have more time to practice the skills and strategies learnt within the program, and may then display improved sadness coping. Another possibility is that the outcome is due to the way that young children express the emotion of sadness. Research suggests that in preschool-aged children, irritability and sadness are correlated such that irritable behaviors may indicate that a child is experiencing emotions of sadness (Leppert et al., 2019). However, it is possible that parent participants within the present study interpreted irritability as resulting from anger rather than sadness, as this is more commonly true for adults and older children (Deveney et al., 2019). Thus, parents of children who exhibited a reduction in irritable behaviors following the “I Spy Feelings” intervention may have reported this change as evidence that their child had improved in their capacity to cope with anger rather than sadness.

It is important to recognize the limitations of the present study in order to consider possible ways that future studies may overcome them. As previously discussed, a significant limitation of the present study is the low internal consistency of the worry coping scale. This low internal consistency limits the extent to which it can be assumed that the scale is a reliable measure of children’s capacity to cope with worry, and findings regarding the worry coping outcomes can be confidently interpreted (Tavakol and Dennick, 2011). The low internal consistency value associated with the worry coping scale may be due to the fact that it comprised only 3 items (Tavakol and Dennick, 2011). Furthermore, the present study’s selection for emotional coping strategies resulted in all coping scales including only 3–5 items each. It is possible that these items did not represent an adequate range of coping skills and strategies. Thus, children who used emotional coping strategies other than those represented in the scales would have been assigned scores which reflected a lower capacity for coping than their actual coping ability. It would, therefore, be beneficial for future studies to utilize a scale which assesses a wider range of coping skills and strategies.

Another limitation of the present study is that children’s emotional coping abilities were assessed solely *via* parent-report methods. While the use of self-report measures would be restricted by factors such as reading ability for children in pre-primary, it could be argued that a more holistic approach would be beneficial in order to optimally assess emotional coping. Bronfenbrenner’s (1977) ecological systems theory states that children belong to multiple microsystems, which each have a significant impact on their development. Therefore, it may be beneficial for future studies to collect data from various microsystems that the child is a part of. For example, as school is a significant microsystem for children (Bronfenbrenner, 1977), it could be useful to obtain reports from teachers to determine whether children demonstrate any changes in their capacity for emotional coping in the classroom which are not noticeable at home. Furthermore, as family/home life is also a significant microsystem for children (Bronfenbrenner, 1977), it may also be beneficial for future studies to run sessions for parents of children in the intervention group, teaching the same emotional coping strategies. This may serve to enhance the effects of the program, by giving parents the ability to support and foster their children’s use of the learned skills and strategies at home. Indeed, other prevention programs which teach emotion regulation strategies to parents as well as children have demonstrated significant intervention effects (Havighurst et al., 2010).

The present study’s follow-up assessment data was collected only 2 months after the “I Spy Feelings” program was delivered. While children of this age develop at a rapid rate, a 2 month period may not be enough time to assess whether the content of the program is being practiced and implemented. Additionally, the present study cannot make any conclusions regarding long-term effects of the program. It would therefore be beneficial for future research to incorporate 6- or 12-month follow-up assessment points in a longitudinal design. Finally, due to undesirable circumstances, the program was only able to run over 5 weeks within the present study rather than 10 weeks, as it is designed to be. This represents another limitation of the study, as it is possible that the compressed learning period did not allow enough time for processing—decreasing the effectiveness of the program. As this was a pilot study, future research will evaluate the program with a longer teaching period and have an opportunity to mitigate the other previously discussed limitations of the present study.

A key strength of the present study was its universal nature. By involving all children from a classroom in the program, there was little opportunity for stigma-related issues to develop, or for high-risk children to be overlooked (Ahlen et al., 2015). This study was also a valuable screening tool for children experiencing emotional difficulties, so that children at risk could be confidentially referred on for psychological assistance. Furthermore, the present study was the first evaluation of an AOP tailored for children of this age, and one of the first to assess the impact of an AOP on emotion regulation. As this study was conducted with students from Catholic schools, future research evaluating the “I Spy Feelings” program should expand into mainstream schools to see if there are any differences in outcomes across the types of schools.

## Conclusion

The major value of the present research is that it provides insight into the short-term effects of the “I Spy Feelings” program regarding children’s capacity to cope with emotions of anger, sadness, and worry. The present findings were mixed, indicating that the program is effective in enhancing children’s capacity to cope with anger but not sadness, though this may be due to certain limitations of the study. Findings also suggested that the program may have positive short-term effects regarding children’s capacity to cope with worry, however lack of internal consistency of the scale used to measure worry coping prevents conclusions from being made. If verified by further research, the present findings may have important implications for future prevention programs designed to teach young children skills and strategies for emotional coping. It is crucial that this research is pursued in order to optimize these programs, and subsequently improve the wellbeing of children, reduce the prevalence of childhood mental disorders, and assist children in managing difficult experiences throughout their lives.

## Data availability statement

The raw data supporting the conclusions of this article will be made available by the authors, without undue reservation.

## Ethics statement

The studies involving human participants were reviewed and approved by Curtin University Human Research Ethics Committee. Written informed consent to participate in this study was provided by the participants’ legal guardian/next of kin.

## Author contributions

AA conceptualized the project, collected and analyzed the data, and was the main author of the manuscript. RR was the supervisor of the main author and assisted her throughout the research process, as well as contributing to the conceptualization of the project and the direction of the manuscript. NB coordinated the wider AOP project, including the data collection process. RK supervised the development of the research design and the analysis of the data. MC and KT assisted in the analysis of the data and the preparation of the manuscript. KM, AM and JP contributed to the writing of the manuscript. All authors contributed to the article and approved the submitted version.

## Funding

This study was supported by the Government of Western Australia Mental Health Commission.

## Conflict of interest

The authors declare that the research was conducted in the absence of any commercial or financial relationships that could be construed as a potential conflict of interest.

## Publisher’s note

All claims expressed in this article are solely those of the authors and do not necessarily represent those of their affiliated organizations, or those of the publisher, the editors and the reviewers. Any product that may be evaluated in this article, or claim that may be made by its manufacturer, is not guaranteed or endorsed by the publisher.
